# How does survodutide work? Plain language review of a potential new medication for obesity and liver disease

**DOI:** 10.1177/17562848261457269

**Published:** 2026-06-05

**Authors:** Jaime P Almandoz, Alexander Dimitri Miras, Jochen Seufert, Elizabeth Paul

**Affiliations:** 1 Division of Endocrinology, University of Texas, Southwestern Medical Center, Dallas TX, USA; 2 School of Medicine, University of Ulster, Derry, UK; 3 Division of Endocrinology and Diabetology, University of Freiburg Medical Center, Faculty of Medicine, University of Freiburg, Freiburg, Germany; 4 Obesity Action Coalition, Tampa, FL, USA

**Keywords:** Plain language review, survodutide, obesity, MASLD, MASH

## Abstract

What is this review about?

This review provides an overview of a medication called survodutide. **Survodutide** is not yet approved for use but is currently being tested to treat people living with **obesity**, as well as those living with **MASH** (metabolic dysfunction–associated steatohepatitis). Obesity is a long-term disease in which extra energy stored as fat by the body begins to have harmful effects on health. MASH is an advanced form of a condition commonly called “fatty liver disease”, or **MASLD** (metabolic dysfunction–associated steatotic liver disease). MASLD causes abnormal amounts of fat to build up in the liver. Obesity and MASH are linked diseases. Both involve problems related to **metabolism**, that is, the way that the body uses and stores energy. People living with obesity and those living with MASH often have other related health conditions at the same time. Improvements in dietary nutrition and increased physical activity are recommended treatments for people living with obesity and MASH. However, interventions such as medication and bariatric surgery are also effective treatments for metabolic dysfunction for those living with these diseases. Such therapies can help people reach their health goals. This review provides an overview of how survodutide works to help treat obesity and MASH. It also describes the key improvements in health status and the side effects experienced by people who took survodutide in clinical trials.

**Click here to view the PDF**

